# Longer shared parental leave is associated with longer duration of breastfeeding: a cross-sectional study among Swedish mothers and their partners

**DOI:** 10.1186/s12887-020-02065-1

**Published:** 2020-04-14

**Authors:** Maria Grandahl, Jenny Stern, Eva-Lotta Funkquist

**Affiliations:** 1 Department of Women’s and Children’s Health, Uppsala University, Akademiska sjukhuset, SE-751 85 Uppsala, Sweden; 2 Department of Health Promoting Science, Sophiahemmet University, Box 5605, SE-114 86 Stockholm, Sweden

**Keywords:** Breastfeeding, Equal health, Infant, Mode of delivery, Parental leave, Partner, Socioeconomics

## Abstract

**Background:**

Breastfeeding is associated with health benefits for both the mother and infant and is therefore important to support; moreover, parental leave is a beneficial factor for breastfeeding. The Swedish parental leave is generous, allowing each parent to take 90 days; additionally, a further 300 days can be taken by either parent. Generally, mothers take 70% of the parental leave days, mainly during the first year. However, breastfeeding duration has declined in the last decade, and it is not known how shared parental leave is associated with the duration of breastfeeding.

**Aim:**

To investigate how parental leave is associated with the duration of exclusive and partial breastfeeding of the infant during the first 12 months after birth. An additional aim was to describe infants’ and parents’ characteristics and mode of birth in association with the duration of exclusive and partial breastfeeding.

**Methods:**

This cross-sectional study was part of the Swedish Pregnancy Planning Study, conducted in Sweden in 2012–2015. The parents were recruited at 153 antenatal clinics in nine counties. In total, 813 couples completed a follow-up questionnaire 1 year after birth. Linear regression models were used to analyse the association between parental leave and the duration of breastfeeding.

**Results:**

Infants were exclusively breastfed for, on average, 2.5 months (range 0–12 months) and partially breastfed, on average, 7 months (range 0–12 months). Most of the parental leave was taken by the mother (mean = 10.9 months) during the infant’s first 12 months, while the partner took 3 months, on average. The parental leave (used and planned) during the infant’s first 24 months were, on average, 21 months. In the multivariate linear regression analysis, mothers’ and partners’ high level of education (*p* < 0.001, *p* = 0.044, respectively), mothers’ higher age (*p* = 0.049), non-instrumental vaginal birth (*p* = 0.004) and longer parental leave for the first 24 months (*p* < 0.001) were associated with longer duration of partial breastfeeding.

**Conclusion:**

The duration of partial breastfeeding was associated with higher parental educational level, higher age, non-instrumental vaginal birth and longer parental leave.

## Background

The Word Health Organisation (WHO) recommends exclusive breastfeeding for 6 months and partial breastfeeding for 2 years or longer [1–3]. Breastfeeding is associated with many health benefits for both the mother and infant [4] and therefore, beneficial for society. If 90% of the new-borns in the United States were breastfed exclusively for 6 months, it would prevent 3340 maternal or child deaths, and save a total of $3 billion in medical costs [5].

In Sweden, the breastfeeding rates peaked in 1996, when 72% of infants were breastfeeding at 6 months and 43% were doing this exclusively. Since then, the breastfeeding rates have declined; in 2017, 63% of infants were breastfeeding at 6 months and 13% were doing this exclusively [6]. The initiation rate of breastfeeding is still high and comparable to many low-income countries [7], but at 12 months, the prevalence is lower in Sweden (16%) than, for example, in the US (27%) or Norway (35%) [4]. The reason for the decline has been discussed vigorously [8], but there is no consensus about causes. Moreover, the decline has been particularly difficult to explain when breastfeeding has been progressing in other high-income countries [9].

Several factors are associated with shorter period of breastfeeding, for instance, being a first-time mother, emotional distress during pregnancy, separation between infant and mother and giving birth by caesarean section [10–12]. There are also differences in the duration of breastfeeding due to sociodemographic factors such as age and socioeconomic status [13]. A recent review found that women in less privileged position, and women with less education have shorter duration of breastfeeding [14]. Similar findings have been reported in a population-based study in Norway [15]. In addition, a Swedish cohort study found that infants whose father had lower education were less likely to be breastfed up to 12 months of age [16]. In Sweden, breastfeeding rates were lower for mothers with disposable incomes in the first three quartiles than in the last quartile [17]. Nonetheless, even though the breastfeeding rates are influenced by socioeconomic status, the decline cannot be explained by the widening socioeconomic gap [18].

The United Nations’ (UN) Sustainable Development Goals commit governments to ensure healthy lives and promote well-being for all [19]. Breastfeeding contributes to most of the goals and the achievement of a more prosperous and sustainable future for people and the planet. Different approaches have been identified for countries to achieve the goals, and one of these is paid parental leave [20]. Studies undertaken in the US, New Zealand and Europe indicate that paid parental leave supports initiation of and the duration of breastfeeding [16, 21–23] and increases exclusive breastfeeding [20]. Few studies have examined the association between paid paternal leave and the duration of breastfeeding. However, a Swedish register-based study found that infants whose fathers took parental leave were breastfed to a higher extent during the first 6 months compared to infants whose fathers had not taken parental leave [16].

Sweden has one of the most generous parental leave programmes in the world, which enables parents to stay at home with their child for a total of 480 days, while receiving up to 80% of their wages from the state. Ninety of these days are reserved for each parent. Statistics show that the mother takes about 70% of the days and the partner 30% and that the number of days taken by the partner is increasing. Most women (83%) take parental leave on full-time basis during the first 12 months or longer. Fifteen per cent of the parents have an equal share of parental leave (at least 40/60). Twenty-five per cent of the fathers take parental leave for 6 months or longer. During the child’s first year, both parents can take parental leave in the same period, for maximum 30 days. The partner also has the right to take 10 days of temporary leave in connection with a child’s birth. As long as the child is under the age of one, parents have the right to full-time parental leave; moreover, until the child is 8 years old, they have the right to work part-time, with or without parental benefits [24].

To the best of our knowledge, how the distribution of parental leave between the parents affects breastfeeding has not been studied previously. Thus, the aim of the present study was to investigate how the duration of exclusive and partial breastfeeding of the infant during the first 12 months after birth is associated with parental leave. In addition, the aim was to describe the infants’ and parents’ characteristics and mode of birth in association with the duration of exclusive and partial breastfeeding.

## Methods

### Study design, sample and procedure

**Fig. 1:**
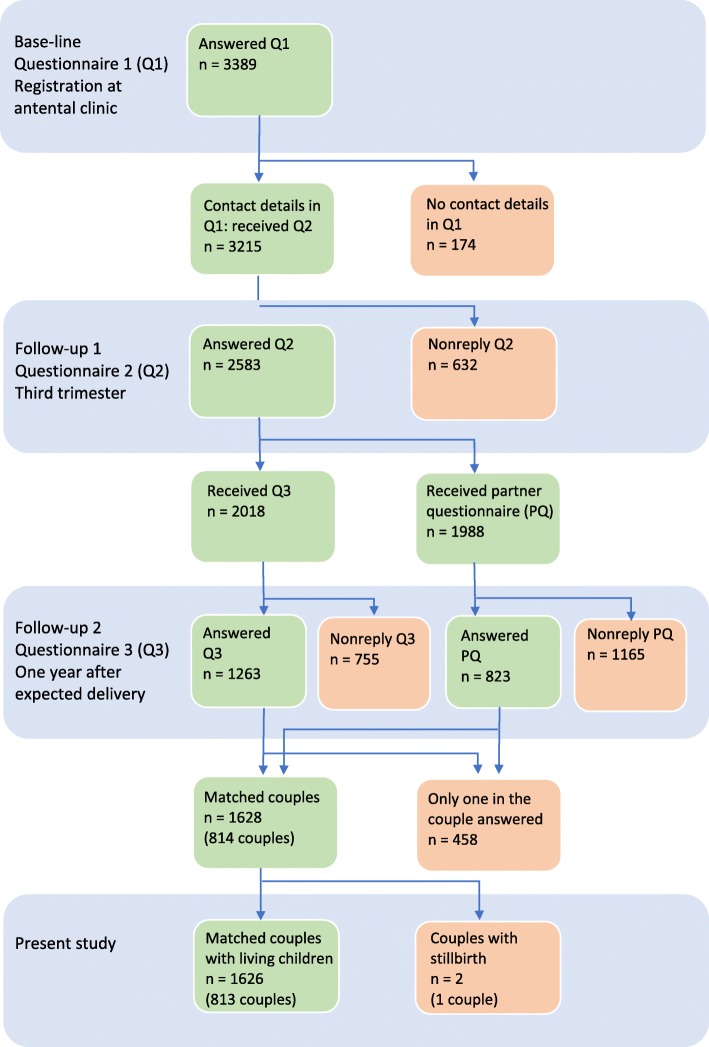
Flowchart of eligible and participant parents

This cross-sectional study was part of a longitudinal project called ‘the Swedish Pregnancy Planning Study’ undertaken in 2012–2015 [25]. Antenatal clinics (*n* = 215) in nine (*n* = 9/21) counties in Sweden were initially invited to participate, and 153 (71%) agreed to participate. Midwives approached women (*n* = 5493) upon registration at the antenatal clinics. Women completed questionnaires (Q) in early pregnancy (*n* = 3389, Q1), third trimester (*n* = 2583, Q2) and one-year post-partum (*n* = 1263, Q3). In connection with Q3, the women were also asked to distribute the Q3 to their partners (Q3p), and 823 partners completed the former. More details regarding the recruitment process are found in Fig. 1 and in Stern et al. [25]. For this study, we excluded participants if only the mother or only the partner had completed Q3, and couples who experienced stillbirth (*n* = 1). The final sample consisted of 813 matched couples (Fig. 1).

### Definitions

We used the WHO definition that describes exclusive breastfeeding as when the infant eats only breast milk, with the supplement of vitamins and/or medications [26]. Partial breastfeeding means that the infant is given infant formula and/or food in addition to breastmilk. Parental leave was defined as time spent at home with the child, with or without parental benefit, and presented for the mother and the partner both separately and jointly.

### The questionnaires

The mothers and their partners answered separate questionnaires 1 year after expected delivery. The questionnaires comprised a total of 65 questions. In the present study, we included data from questions about demographic background characteristics (age, sex, previous children, country of birth, level of education and household income), about the pregnancy (level of pregnancy planning, single/multiple pregnancy), mode of delivery (how it started, ended and if there was haemorrhage > 1000 ml), and about the infant (birth weight, gestational age, sex, neonatal care, congenital states and twins). The mothers and partners both reported their own use of parental leave, with or without parental benefit, for the first 12 months after delivery. Both parents also reported their planned use of parental leave for 24 months after birth. In addition, the mothers answered items about how the infant was fed during the first 12 months. For each month, they were asked to tick all of the following that applied to them: breast milk, infant formula, tiny little tastes (1 ml), small samples (1–2 teaspoons) and food (> 1 tablespoon), see Additional file 1 for more details.

### Statistical analyses

The Statistical Package for the Social Sciences version 25.0 (SPSS Inc., Chicago, Illinois, USA) was used for the statistical analyses. Sample characteristics are presented by descriptive statistics*.* Parental leave was operationalised as three measures at two different time periods, see Table 1. The association between the mother’s and partner’s parental leave was analysed using Pearson’s correlation.

**Table 1:** Operationalisations of parental leave

	*Used* weeks of parental leave during the first 12 months after birth	*Used* and *planned* weeks of parental leave during the first 24 months after birth
Mother’s parental leave	MPL12	MPL24
Partner’s parental leave	PPL12	PPL24
Total parental leave	TPL12	TPL24

A linear regression was used to analyse the effect of background variables and parental leave (independent variables) on the duration of both exclusive and partial breastfeeding (dependent variables), respectively. Independent variables were chosen based on previous knowledge on breastfeeding [10–12, 14, 16, 20].

Independent variables on nominal and ordinal level were dichotomised as follows:

- level of education (lower than university education versus university education)
- country of birth (Sweden versus other/do not know)
- household income/month (low (< 40,000 SEK/4000 EUR) versus average/high (> 40,000 SEK/4000 EUR)
- planned pregnancy (highly/quite planned versus quite/highly unplanned)
- previous children (no versus yes)
- neonatal care (no versus yes)
- congenital states in need of care (no versus malformation/injury/disease)
- mode of birth: start of delivery (spontaneous versus induced/planned caesarean section)
- mode of birth: end of delivery (normal versus instrumental/caesarean section or complications)

Independent variables were analysed at univariate level, and all significant variables were then included in the analysis at multivariate level. If both *the mothers and/or the partners used and planned use of parental leave* and *the total used and planned use of parental leave* was significant at univariate level, only *the total* was included in the multivariate model to avoid overlapping independent variables. A *p*-value of < 0.05 was considered significant.

## Results

### Sample and characteristics

Mothers who participated in the study were between 19 and 49-years-old, and partners between 22 and 57-years-old, see Table 2. Characteristics about the pregnancy, mode of birth and child are presented in Table 2.

**Table 2:** Background characteristics of the mother, partner, pregnancy, mode of birth and child

***Characteristics of study sample***	***Study sample*** ***Mean (SD)***	***Study sample*** ***Frequency (%)***	***Comparison*** ***Official Statistics Sweden***
Mother			
Age, years	31.7 (4.6)		30.3^a^
Born outside Sweden		73 (9)	27.5^b^
University education		475 (58)	49^b^
Previous children		415 (51)	56^a^
Partner			
Age, years	34.3 (5.5)		34.0^a^
Sex: female		14 (2)	d
Born outside Sweden		64 (8)	11.6
University education		349 (43)	38
High household income		510 (63)	550^e^
Pregnancy			
Level of pregnancy planning			
Highly planned		429 (52)	d
Quite planned		215 (26)	d
Neither planned nor unplanned		92 (11)	d
Quite unplanned		28 (3)	d
Highly unplanned		47 (6)	d
Single pregnancy		792 (97)	98^b^
Multiple pregnancy		5 (0.6)	1.4^b^
Mode of delivery			
Spontaneous vaginal		614 (76)	83^b^
Induced vaginal		132 (16)	16.7^a^
Planned Caesarean		60 (7)	8^b^
Complications			
Haemorrhage^g^ (> 1000 ml)		57 (7)	7.2^c^
Emergency Caesarean		66 (8)	8^a^
Instrumental delivery		65 (8)	7.2^a^
Infant			
Birth weight, grams	3585 (550.5)		3565^b^
Gestational age, weeks	39 (1.6)		39-40^b^
Sex			
Girl		401 (49.4)	48.6^b^
Boy		410 (50.5)	51.3^b^
Neonatal care		46 (6)	~ 10 ^b,e^
Congenital state in need of care			
Malformation		12 (1)	2-3^a^
Injury		2 (0.2)	
Disease		19 (2)	3.7/1000^a^

^**a**^ The National Board of Health and Welfare

^b^ Statistics Sweden

^c^ Vaginal (section = 10.4%)

^d^ No reliable data available

^e^ 5.5% gestation week < 37)

^f^ Children born with malformation or injury at birth

^g^ Bleeding before, during or after the delivery

### Parental leave

The mothers took, on average, 10.9 months of parental leave during the first year after birth (M: 43.7 weeks, SD: 7.4, range: 0–48). The corresponding figure for their partners was 3.0 months (M: 11.6 weeks, SD: 10.0, range: 0–48). The total use of parental leave during the first year after birth was, on average, 13.8 months (M: 55.4 weeks, SD: 9.8, range: 0–96). The mothers planned to take, on average, 4.0 months of parental leave during the second year after birth (M: 16.0 weeks, SD: 13.6, range: 0–12) and their partners 3.4 months (M: 13.5 weeks, SD: 10.8, range: 0–48). The average total use of and planned use of parental leave during the 24 months after birth was 21.0 months (M: 84.2 weeks, SD: 21.4, range: 0–192).

**Fig. 2:**
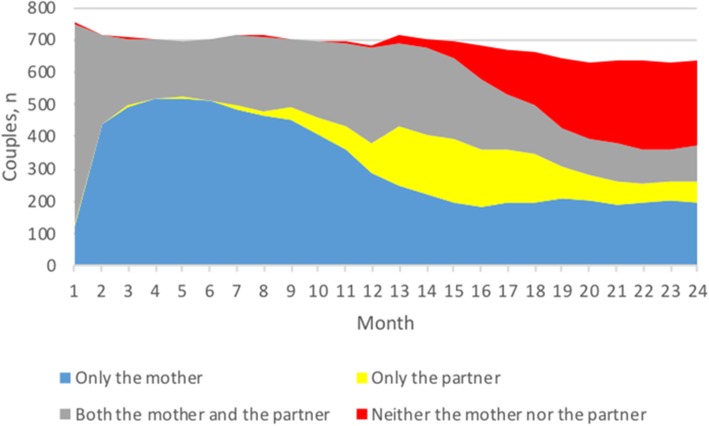
Used and planned use of parental leave for the first two years after birth, with or without parental benefit.

Figure 2 illustrates the used and planned use of parental leave for the first 24 months after birth. The mothers’ and partners’ total parental leave during the first 24 months (used and planned) was negatively correlated (*r* = − 0.85, *p* < 0.001). Thus, the more parental leave used or planned by the partner, the less parental leave used or planned by the mother.

### Breastfeeding

Infants were exclusively breastfed for, on average, 2.5 months (range 0–12) and breastfed to any extent, on average, 7 months (range 0–12).

### Factors associated with the duration of exclusive breastfeeding

Variables associated with exclusive breastfeeding on univariate level were included in the multiple regression model and are presented in Table 3. In the multivariate linear regression analysis, higher maternal education, previous births, singleton pregnancy, normal delivery (start and end of delivery) and longer parental leave (TPL24) were associated with longer duration of exclusive breastfeeding (Table 3). The following variables were not associated with the duration of exclusive breastfeeding in the linear regression analysis on univariate level: partner’s level of education, household income, partner’s age, mother’s country of birth, partner’s country of birth, level of pregnancy planning, birth weight, sex of the infant, neonatal care, congenital state of the infant, mother’s use of parental leave (MPL12), partner’s use of parental leave (PPL12), total use of parental leave (TPL12), and partner’s used and planned use of parental leave (PPL24) (data not shown).

**Table 3:** Results for univariate and multivariate linear regression models with the duration of exclusive breastfeeding during the first 12 months after birth as the outcome

	Univariate regression				Multiple regression^**a**^	
Variables	***R Square***	***Adjusted R Square***	***Beta-coefficient***	***p***	***Beta-coefficient***	***p***
Mother’s level of education (low/high)	0.020	0.018	2.145	< 0.001	1.723	0.004
Mother’s age	0.011	0.010	0.171	0.003	0.060	0.391
Gestational age	0.006	0.004	0.341	0.034	0.299	0.089
Previous children	0.021	0.019	2.152	< 0.001	1.826	0.002
Pregnancy, single versus multiple	0.013	0.012	−11.034	0.001	−10.022	0.045
Mode of birth/start of delivery (spontaneous versus other)	0.027	0.025	−2.870	< 0.001	− 2.001	0.006
Haemorrhage in connection with delivery	0.008	0.007	−2.634	0.011	− 1.639	0.133
Mode of birth/end of delivery (normal versus instrumental/ caesarean)	0.023	0.022	−2.962	< 0.001	− 1.919	0.011
The mother’s used and planned use of parental leave for the first 24 months	0.005	0.004	0.032	0.038	–	–
Total used and planned use of parental leave for the first 24 months	0.007	0.006	0.030	0.016	0.038	0.003

^*a*^*Model summary R*^*2*^ *= 0.094 Adjusted R*^*2*^ *= 0.082*

### Factors associated with the duration of partial breastfeeding

Variables associated with partial breastfeeding on univariate level were included in the multivariate model and are presented in Table 4. In the multivariate linear regression analysis, higher level of mothers’ and fathers’ education, higher maternal age, normal delivery (normal versus instrumental/caesarean section or complications) and longer parental leave (TPL24) were associated with the duration of any breastfeeding (Table 4). The following variables were not associated with the duration of any breastfeeding in the linear regression analysis on univariate level: mother’s country of birth, partner’s country of birth, level of pregnancy planning, birth weight, sex of the infant, neonatal care, congenital state of the infant and haemorrhage in connection with delivery, mother’s use of parental leave (MPL12), partner’s use of parental leave (PPL12), total use of parental leave (TPL12), mother’s used and planned use of parental leave (MPL24) (data not shown).

**Table 4:** Results for univariate and multivariate linear regression models with the duration of any breastfeeding during the first 12 months after birth as the outcome

	Univariate regression				Multiple regression^**a**^	
Variables	***R Square***	***Adjusted R Square***	***Beta-coefficient***	***p***	***Beta-coefficient***	***p***
Mother’s level of education (low/high)	0.066	0.065	7.553	< 0.001	5.005	< 0.001
Partner’s level of education (low/high)	0.035	0.033	5.406	< 0.001	2.340	0.044
Household income (low/high)	0.013	0.012	3.264	0.001	0.077	0.945
Mother’s age	0.053	0.052	0.716	< 0.001	0.320	0.049
Partner’s age	0.029	0.028	0.443	< 0.001	0.157	0.198
Gestational age	0.008	0.006	0.773	0.013	0.581	0.081
Single/multiple pregnancy	0.012	0.010	−19.381	0.002	−13.379	0.155
Mode of birth/start of delivery (spontaneous versus other)	0.011	0.010	−3.584	0.003	− 1.373	0.314
Mode of birth/end of delivery (normal versus instrumental/caesarean)	0.014	0.012	−4.352	0.001	− 3.930	0.004
The partner’s used and planned use of parental leave for the first 24 months	0.020	0.019	0.124	< 0.001	–	–
Total used and planned use of parental leave for the first 24 months	0.022	0.021	0.102	< 0.001	0.097	< 0.001

^*a*^*Model summary R*^*2*^ *= 0.143 Adjusted R*^*2*^ *= 0.130*

## Discussion

The majority of both used and planned parental leave was taken by the mother. However, the more parental leave taken or planned by the partner, the less parental leave was taken or planned by the mother. Infants were exclusively breastfed for, on average, 2.5 months; moreover, the duration was associated with mothers’ level of education, previous children, multiple pregnancy, mode of delivery (start and end) and total used and planned use of parental leave. Regarding the duration of partial breastfeeding, associated factors were mothers’ and partners’ level of education, mother’s age, end of delivery and total used and planned use of parental leave. Consequently, mothers with higher level of education, higher age, normal end of delivery and living in a family with longer use of total parental leave had a longer duration of partial breastfeeding.

Breastfeeding is more than a choice; it is an investment in future health for both the mother and infant [27] and contributes to achieving many of the UNs 17 Sustainable Development Goals. Breastfeeding is linked to factors such as economy, health outcomes, sustainable consumption, gender equality and workplace rights [19]. The United Nations has pointed out paid parental leave as an important approach for countries in order to strengthen mothers’ opportunity to breastfeed [20]. Several studies support this approach, since parental leave supports initiation of and the duration of breastfeeding [16, 21–23] and increases exclusive breastfeeding [20]. Maternal leave is frequently cited as a facilitator for breastfeeding [14], but paternal leave has also shown, in a Swedish study, to have positive impact on breastfeeding [16].

In our study, there was no association between parental leave during the first year and the duration of breastfeeding (exclusive or partial), either for the mother, partner or their total leave. It is not surprising that since almost all Swedish children stay at home with one parent during their first year, parental leave is not a decisive factor for breastfeeding in this setting. However the mothers’ total parental leave was associated with the duration of exclusive breastfeeding and the partners’ total parental leave with the duration of partial breastfeeding. The mothers’ and partners’ total parental leave during the first 2 years was associated with both exclusive and partial breastfeeding during the first year, also after adjusting for background factors, suggesting that the longer the total parental leave, the longer the duration of breastfeeding.

Support from the partner is an important factor for successful breastfeeding [28]; moreover, the partners’ use of parental leave can be seen as an expression of support in caring for the child. Previous research has, for instance, shown that the longer the father was present at the ward after delivery, the longer the first-time mother breastfed [29]. However, the question is complicated by the fact that one should distinguish between practical and emotional support, and that practical support from the partner could be a barrier to breastfeeding in high-income countries [30]. Interestingly, in the present study, we found no indication that partners’ use of parental leave could be a barrier to breastfeeding. On the contrary, the longer the total used and planned use of parental leave, the longer the duration of total breastfeeding. Paid parental leave both facilitates parents and infant’s relationship and promotes breastfeeding [21]. Furthermore, it improves overall child health and maternal mental health [31]. Partners’ increased parental leave also highlights the need for partners to gain greater knowledge on how to support breastfeeding [32].

While breastfeeding is increasing in several countries [4], Sweden shows decreasing trend of breastfed infants in the most recent 10 years [8]*.* Consequently, the duration of time that mother’s breastfed exclusively in this study was significantly lower than the recommendation of 6 months by WHO. The reasons for this might be multifactorial [10, 11, 27, 33]. Certain changes in Sweden in recent decades may have affected mothers who want to breastfeed and contributed to less incidences of breastfeeding. The proportion of caesarean sections as a mode of birth has increased in Sweden since the 1990s, from about 10 to 18% [34]. It is well known that mode of delivery is an important factor associated with successful breastfeeding initiation and duration [11]; moreover, a meta-analysis has reported negative association between planned caesarean section and early breastfeeding [12]. Thus, it is not surprising that mode of delivery was associated with the duration of breastfeeding, while spontaneous vaginal births resulted in more breastfeeding. Complicated deliveries might lead to disruption of the infant/mother dyad and thereby decrease initiation of breastfeeding. The Baby-friendly Hospital Initiative (BFHI) implemented by UNICEF and WHO is a successful and evidence-based programme to avoid separation and to support breastfeeding [27]. During the 1990s, Sweden was one of the countries that took a leading role in the implementation of BFHI, and 97% of all maternity care facilities were designated as baby-friendly in order to protect, promote and support breastfeeding [6]. However, the responsibility of meeting the standards of the BFHI is no longer supervised in Sweden [26]. In order to deal with the decreasing trend in breastfeeding in Sweden, the programme needs to be a carefully re-evaluated. Breastfeeding support must be a government priority, with an official body in charge of maintaining the BFHI standards.

The multivariate linear regression analysis showed that maternal factors such as high level of education and previous children were positively associated with the duration of exclusive breastfeeding. In addition, high maternal education level was positively associated with the duration of partial breastfeeding. This reaffirms previous research findings that mothers with high level of education and mothers with previous children are more likely to breastfeed [10, 35]. Previous breastfeeding experience improves the ability to breastfeed, and parents with high level of education might have more flexibility regarding use of parental leave days, especially during the child’s second year [36]. This might be a facilitating factor, resulting in longer duration of breastfeeding. Short or no breastfeeding may also be due to factors related to the infant. In the present study, it turned out that multiple pregnancy, i.e. twins, was a barrier to breastfeeding. This is also in line with previous research [37].

The duration of breastfeeding is a matter of equity and equal health among present and future generations. Promotion of breastfeeding in a high-income society such as Sweden is in line with the UNs and WHOs global goals and in the best interest of the individual child as well as overall public health. Consequently, parental leave might be one facilitating factor for successful breastfeeding in high-income societies. However, this subject is still quite unexplored. Future research is needed to understand why neither maternal nor partner parental leave during the first 12 months were associated with the duration of breastfeeding. We propose the use of qualitative research to explore how the duration of individual parental leave might be less relevant to breastfeeding than the total duration, as well as what factors parents believe are important for breastfeeding (exclusive and partial).

### Strengths and limitations

This study is the first study investigating parental leave and the duration of breastfeeding among Swedish parents. The study provides data for a large number of Swedish parents (*n* = 1626), and the sample represents a wide geographical area, including both rural and urban areas with both high and low socioeconomic statuses.

The participants completed the questionnaires thoroughly; thus, the internal missing data was low. In addition, the items measuring breastfeeding duration (exclusive and partial) are very detailed and thereby probably more reliable than the Swedish register-based data [6], explaining the low duration in this study. However, the response rate for the present study is lower in comparison to the baseline data collection. It was challenging to collect data among the partners as we did not have any personal data on them. Consequently, we had to go through the participating women, and only 823 of 1988 eligible partners completed the partner questionnaire (Q3). Therefore, we could only match 813 couples from the initial cohort of 3389 pregnant women (Fig. 1). In addition, there might be a selection bias since the sample mainly includes Swedish-born parents. This is unfortunately common in research in general and similar studies among parents in particular. Furthermore, self-reported data should always be interpreted with caution. Even if the cross-sectional design cannot provide cause and effect, we used robust statistical analyses. Thus, we believe that the results might be representative of parents in similar contexts. In order to avoid too small subgroup analyses, we have categorised mode of delivery into normal versus instrumental/caesarean. This might be a strength as well as a limitation.

## Conclusion

This is the first study investigating whether there is an association between mothers’ and partners’ duration of parental leave and exclusive and partial breastfeeding. The duration of exclusive breastfeeding was associated with mothers’ level of education, previous children, multiple pregnancy, mode of delivery (start and end) and total used and planned use of parental leave. Our findings also indicate that there is an association between the duration of partial breastfeeding and mothers’ and partners’ level of education, mothers’ higher age, end of delivery, and parents’ total used and planned use of parental leave.

## Supplementary information

**Additional file 1.** Supplemental material Questionnaire, item breastfeeding and food first 12 months.

## Data Availability

The datasets generated and analysed during the current study are not publicly available due to the risk of identifying participants but are available upon reasonable request. Principle Investigator for the Swedish Pregnancy Planning Study (SWEPP), Dr. Maria Jonsson (maria.jonsson@kbh.uu.se), Department of Women’s and Children’s Health, Uppsala University, Uppsala Sweden.

## ABBREVATIONS

BFHI: Baby-Friendly Hospital Initiative.
UN: United Nation.
WHO: World Health Organisation.
