# Supplementary material for: Longer shared parental leave is associated with longer duration of breastfeeding: a cross-sectional study among Swedish mothers and their partners
Source: BMC Pediatr. 2020 Apr 14;20:159. doi: 10.1186/s12887-020-02065-1 (PMC7155253; doi:10.1186/s12887-020-02065-1)
Supplement: Supplementary file 1 — Additional file 1. Supplemental material Questionnaire, item breastfeeding and food first 12 months. [file 12887_2020_2065_MOESM1_ESM.docx]

Additional file 1

Item measuring the child’s food

Vad har ditt barn fått för mat under sitt första år? Kryssa för alla alternativ som stämmer vid varje levnadsmånad. Flera alternativ kan ges under samma tidsperiod.


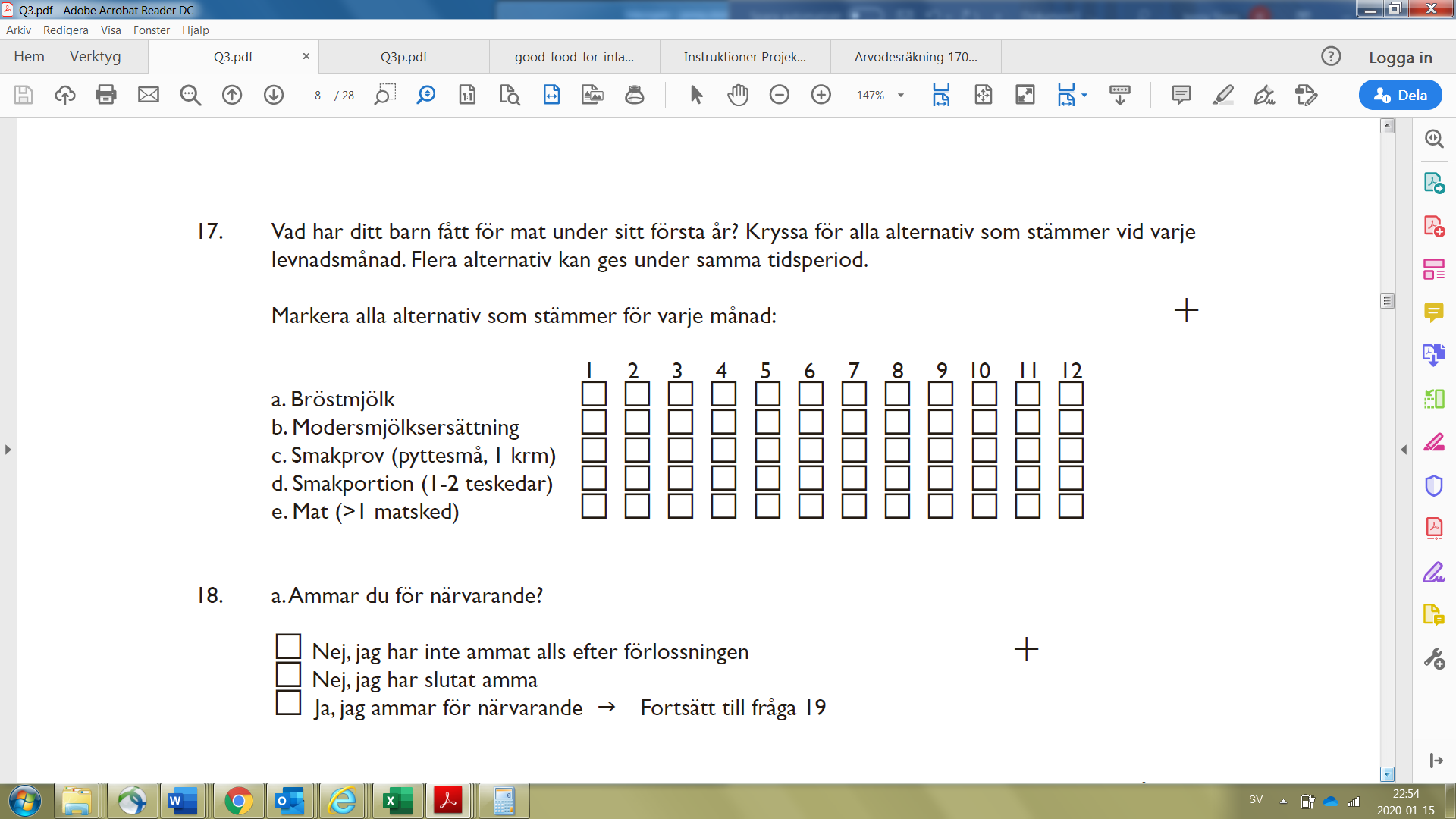
Markera alla alternativ som stammer för varje månad:

1. Bröstmjölk
2. Modersmjölksersättning
3. Smakprov (pyttesmå, 1 krm)
4. Smakportion (1-2 teskedar)
5. Mat (>1 matsked)

What kind of food did your child get during his/her first year? Tick all options that applies to your child for each month. Several options can be ticked for each month.

Select all options that applies for each month:
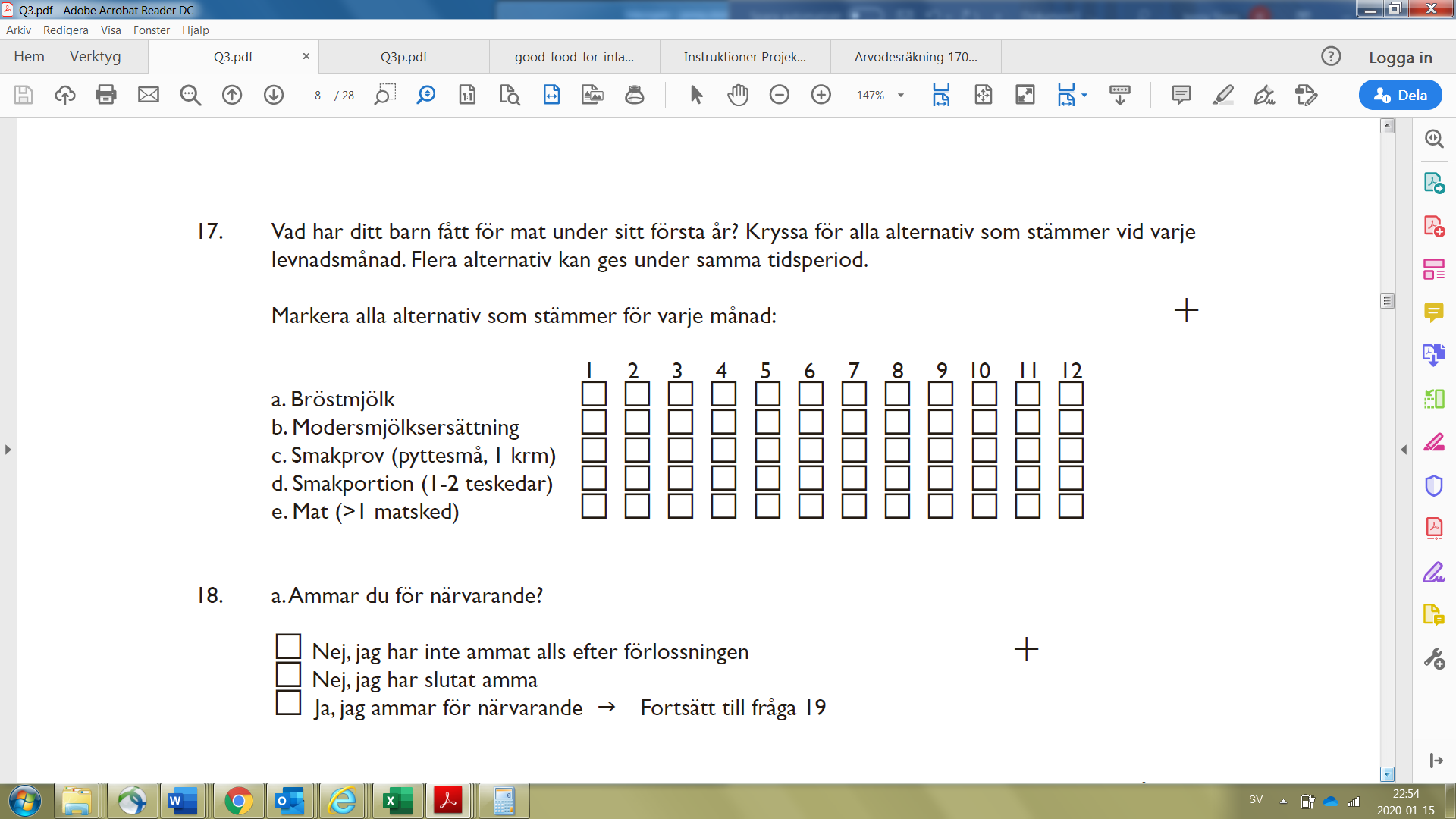


1. Breast milk
2. Infant formula
3. Tiny little tasters (1 ml)
4. Small samples (1-2 teaspoons)
5. Food (> 1 tablespoons)
